# Diagnosis of benign notochordal cell tumor of the spine: is a biopsy necessary?

**DOI:** 10.1002/ccr3.1287

**Published:** 2017-11-24

**Authors:** Satoshi Tateda, Ko Hashimoto, Toshimi Aizawa, Haruo Kanno, Shin Hitachi, Eiji Itoi, Hiroshi Ozawa

**Affiliations:** ^1^ Department of Orthopaedic Surgery Faculty of Medicine Tohoku Medical and Pharmaceutical University Sendai Japan; ^2^ Department of Orthopaedic Surgery Tohoku University School of Medicine Sendai Japan; ^3^ Department of Diagnostic Radiology Tohoku University School of Medicine Sendai Japan

**Keywords:** Benign notochordal cell tumor, chordoma, magnetic resonance imaging, notochordal rest, spinal tumor

## Abstract

Benign notochordal cell tumor is a benign intraosseous lesion, demonstrates characteristic imaging features. The lesion demonstrates low‐signal intensity in T1‐weighted images, high‐signal intensity in T2‐weighted images, and no enhancement with contrast medium in MRI and slight osteosclerosis in CT. If typical imaging findings are identified, biopsy is not necessary.

## Introduction

Benign notochordal cell tumor (BNCT) is a benign intraosseous lesion derived from notochordal cells. They are very common lesions, with a reported prevalence of 20% in autopsy findings 1. In recent years, because of the increasing availability of magnetic resonance imaging (MRI), the number of BNCT lesion identified as an abnormal finding is expected to rise. The prevalence of BNCT detected during imaging was reported to be 0.76% 2. BNCT has characteristic imaging features on computed tomography (CT) and MRI, and the opportunities to visualize this disease should be increasing. However, the diagnosis of this disease may be difficult because BNCT is not yet widely recognized.

Here, we report the clinical features of two BNCT cases wherein the diagnoses were confirmed by histopathological investigation following biopsy.

## Case Reports

### Case 1

**Figure 1:**
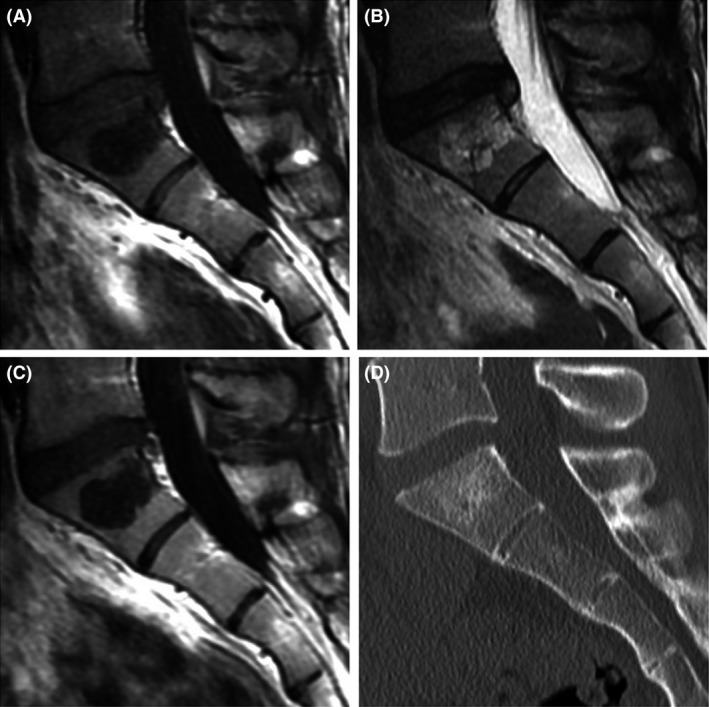
Imaging findings in case 1. On MRI of the sacral spine, the tumorous lesion has clear margins and is enclosed by the S1 vertebral body. The signal intensities are homogeneously low in T1‐weighted images (A) and high in T2‐weighted images (B), and no enhancement is observed in T1‐weighted images with a gadolinium contrast medium (C). No extraosseous lesion or enlargement is observed. CT image demonstrates slight osteosclerosis throughout the tumorous lesion and no osteolytic changes in the vertebral body (D).

**Figure 2:**
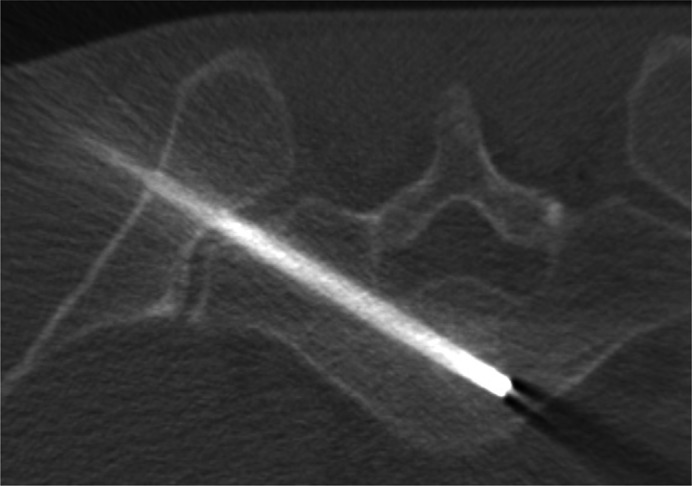
An image of CT‐guided needle biopsy procedure in case 1. The vertebral tumor has been accessed through the sacroiliac joint to obtain a biopsy specimen.

**Figure 3:**
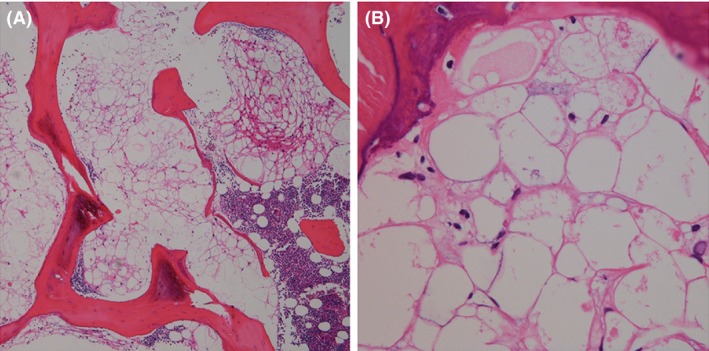
Histopathological findings in case 1 (H&E, A ×4, B ×40). Photomicrographs show proliferation of physaliferous cells without nuclear atypia between thickened bone frameworks. There was no bone destruction and myxoid matrix.

A 22‐year‐old male was referred to our university hospital for the investigation of a sacral lesion detected on lumbar spine MRI at a local orthopedic clinic. His primary symptom was lower back pain upon movement. MRI of the sacral spine revealed an intraosseous lesion with clear margins enclosed by the S1 vertebral body. The signal intensities of the lesion were homogeneously low in T1‐weighted images and high in T2‐weighted images, and no enhancement was observed in T1‐weighted images with a gadolinium contrast medium. There were no extraosseous lesions or enlargements (Figure 1A−C). CT demonstrated a slight osteosclerosis throughout the tumorous lesion. There were no osteolytic changes in the vertebral body (Figure 1D). On positron emission tomography (PET), no uptake of radioisotope‐labeled glucose was observed in the tumorous mass. Because no additional abnormalities were identified on whole‐body analysis, a CT‐guided needle biopsy of the vertebral tumor was performed (Figure 2). The histopathological findings revealed that the tumor consisted of physaliferous cells without nuclear atypia or myxoid matrices. The trabecular bone showed slight thickening, and no bone destruction was identified. The tumor cells stained positive for AE1/AE3 and EMA and negative for S100 and Brachyury. The percentage of cells that were positive for Ki‐67 was less than 1% (Figure 3). The lesion was diagnosed as BNCT, and periodic observation without invasive tests was chosen as the course of treatment.

### Case 2

**Figure 4:**
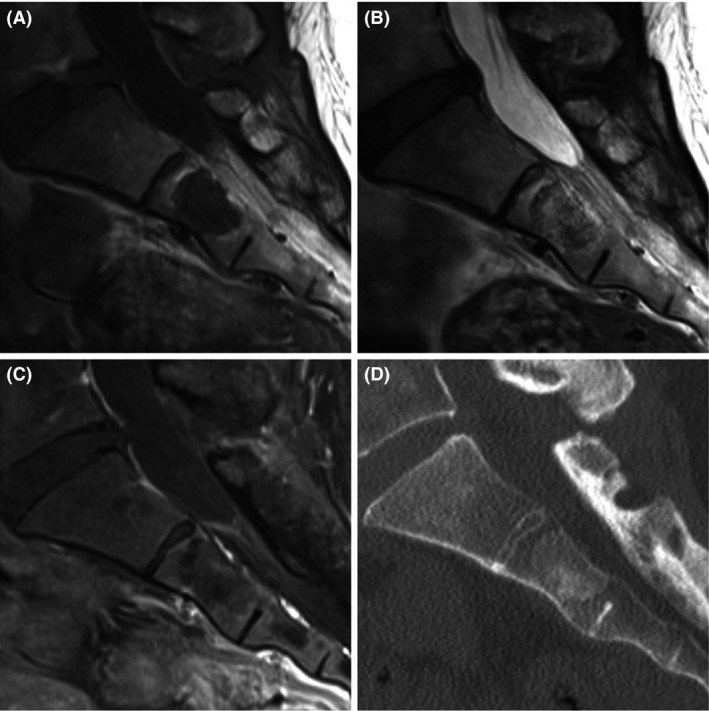
Imaging findings in case 2. On MRI of the sacral spine, the signal intensities of the tumorous lesion are homogeneously low in T1‐weighted images and high in T2‐weighted images, and no enhancement is observed in fat‐suppression T1‐weighted images with a gadolinium contrast medium in the S2 vertebral body (A−C). CT image demonstrates slight osteosclerosis throughout the tumorous lesion and no osteolytic changes (D).

A 42‐year‐old female was referred to our hospital for further investigation of a sacral tumor identified on pelvic MRI at a gynecologic clinic. The patient was asymptomatic and had no abnormal physical findings. MRI revealed that the signal intensities of the tumorous lesion were homogeneously low in T1‐weighted images and high in T2‐weighted images, and no enhancement was observed in fat‐suppression T1‐weighted images with a gadolinium contrast medium (Figure 4A−C). CT of the lesion demonstrated slight osteosclerosis without osteolytic changes (Figure 4D). The histopathological investigation following by CT‐guided needle biopsy revealed that the lesion was BNCT. Periodic follow‐up was selected as the course of treatment for this patient also.

## Discussion

BNCT is a benign intraosseous lesion derived from notochordal cells. BNCT was first reported by Darby et al. 3. and termed an “intraosseous chordoma.” Following the publication of case reports by other investigators 4, 5. Yamaguchi et al. reported the prevalence of BNCT in autopsy findings, advocating the use of the term “benign notochordal cell tumor” in 2004 1. According to their report, BNCT is a very common pathological lesion because it was found in approximately 20% of the autopsy findings, ranging from 1 mm to 13 mm in size. The common sites of BNCT were reported to be the sacrum and clivus; however, it has been frequently found in the cervical and lumbar region of the spine in clinical situations. In recent years, an increasing number of BNCT lesions have been identified as abnormal findings on spinal MRI scans. Golden et al. examined the posterior clivus of 916 patients using CT and MRI and reported the imaging prevalence of BNCT to be 0.76% 2.

The common sites of BNCT are similar to those of chordoma. In rare cases, there are some reports that BNCT coexists with chordoma. Some reports suggested that BNCT may serve as a precursor lesion of chordoma 6, 7, 8, 9. In contrast, other investigators disagreed with this hypothesis because of little evidence 10. Meanwhile, the relationship between chordoma and BNCT remains to be elucidated. With respect to the natural course of BNCT, Mirra et al. 4 reported no enlargement of a BNCT lesion after 9 years of natural course. Iorgulescu et al. 10 also reported no progression of the lesion in eight patients after an average follow‐up of 21.6 months. No case of a diagnosed BNCT transforming into a chordoma has been reported. The current preferred choice of treatment is periodic follow‐up without any specific treatment 11.

The imaging findings of BNCT reported characteristic as shown in our cases 10, 11, 12. According to these reports, on MRI, the lesion has a clear margin with internal homogeneous signal intensity; low‐signal intensity in T1‐weighted images, high‐signal intensity in T2‐weighted images, and a lack of enhancement with gadolinium contrast medium. The lesion was localized in intraosseous sites only, and no extraosseous lesion was observed 10, 11, 12. On CT, BNCT lesions demonstrate slight intraosseous sclerotic changes 10, 11, 12. Histopathological analysis of BNCT specimens usually shows preserved trabecular patterns in the vertebral body. The slight intraosseous sclerotic changes on CT indicate slight thickening of the remaining trabecular bone. Nishiguchi et al. 12 reported BNCT and chordoma can be distinguished by images from its characteristic image findings. However, the observation of extraosseous invasion, osteolytic changes, or destruction of the vertebral cortex suggests a malignant tumor, such as chordoma, as indicated in the literature 11. Surgical excision is recommended for tumors with extraosseous or osteolytic lesions, even when needle biopsy results are suggestive of BNCT 10.

In image findings, there are several diseases that need to be distinguished from BNCT. (Table 1) In MRI, malignant vertebral tumors demonstrate low‐signal intensity in T1‐weighted images, high‐signal intensity in T2‐weighted images, and clear enhancement with gadolinium contrast medium, which reflects hypervascularity in the tumor. Morphologically, various patterns can be seen such as irregularities of the tumor margin, irregularities of the internal structures reflecting necrotic tissues, and extraosseous extension of tumor lesion 13, 14. CT scans usually reveal osteolysis or osteosclerosis in the malignant vertebral tumors 13, 14, 15. Hemangioma, a common benign vertebral tumor, demonstrates high‐signal intensities in T1‐ and T2‐weighted images, remarkable enhancement on MRI, and a typical “polka‐dot sign” on CT, indicating thickened trabeculae in the vertebral body 16. On BNCT, the lesion demonstrates low‐signal intensity in T1‐weighted images, high‐signal intensity in T2‐weighted images like malignant tumor. However, the tumor is confined within the vertebral body, and as a characteristic finding, the lesion is not enhanced with gadolinium contrast medium on MRI. The image finding is very specific that is not enhanced at all, with the low‐signal intensity in T1‐weighted image and high‐signal intensity in T2‐weighted images in MRI. In the case of malignant tumor, it will be enhanced to some extent. If similar findings are found with MRI, it is about cystic lesions. In addition, CT confirms characteristic osteosclerotic images, which enable more reliable diagnosis. When these image findings are complete, it can be diagnosed as BNCT by only images. In our cases, the characteristic findings were detected on MRI and CT scans, and the diagnosis was confirmed on histological examination of the biopsy samples. Considering these findings, BNCT has unique imaging findings on both MRI and CT that can be used for diagnosis with imaging only and without biopsy.

**Table 1:** Differential diagnosis of image findings of vertebral tumor

Tumor	Location	T1WI	T2WI	Enhancement	CT
Malignant tumor	Intraosseous–extraosseous	Low	Low–high	Positive	Osetosclerotic or Osteolytic
Hemangioma	Intraosseous–extraosseous	Low–high	High	Positive	Polka‐dot sign
Benign notochordal cell tumor	Intraosseous only	Low	High	Negative	Osteosclerotic

BNCT is a relatively new type of benign tumor that is not yet widely recognized, although it may be commonly encountered because of the increasing availability of spinal MRI scans. When malignant tumor is suspected in image findings, a needle biopsy should be performed without hesitation. However, needle biopsy of the spine is also accompanied by risk of complications. If it can be determined with characteristic image finding as a benign tumor, it will not bear the unnecessary risk of needle biopsy. Our group no longer performs needle biopsy procedures for patients with typical BNCT characteristics after encountering the cases described above. Knowledge of these typical BNCT characteristics is beneficial to avoid unnecessary expense and invasive procedure in such patients.

## Authorship

ST: performed conception and design, and wrote the manuscript. KH: wrote parts of manuscript and reviewed the manuscript, and performed patient examination. TA and HK: performed patient examination. SH: performed patient examination and prepared radiological images. EI and HO: reviewed the manuscript.

## Conflict of Interest

None declared.
